# Sociodemographic and health-related factors associated with exclusive breastfeeding in 77 districts of Uganda

**DOI:** 10.1186/s13006-023-00604-x

**Published:** 2023-12-05

**Authors:** Derrick Kimuli, Florence Nakaggwa, Norah Namuwenge, Rebecca N. Nsubuga, Paul Isabirye, Kenneth Kasule, Justine Fay Katwesige, Sheila Nyakwezi, Solome Sevume, Norbert Mubiru, Barbara Amuron, Daraus Bukenya

**Affiliations:** 1 Social & Scientific Systems, Inc., a DLH Holdings company / United States Agency for International Development Strategic Information Technical Support Activity, Kampala, Uganda; 2 The United States Agency for International Development Uganda, US Mission Compound - South Wing, Kampala, Uganda

**Keywords:** Exclusive breastfeeding, Discrepancy, Community surveys

## Abstract

**Background:**

Uganda surpasses many African nations and the global average in exclusive breastfeeding (EBF) rates. Yet, malnutrition is a critical issue, with stunting impacting roughly 29% of children under 5 years. Enhancing EBF could mitigate such nutritional challenges. This study focused on determining the current EBF prevalence and identifying associated factors across 77 surveyed districts.

**Methods:**

Pooled data from the Lot Quality Assurance Sampling (LQAS) surveys conducted in 77 districts in Uganda during 2021 and 2022 were analyzed. The analysis involved 7,210 mothers of children under 6 months, EBF was considered as the proportion of infants who received breast milk only in the 24 hours before the survey. A mother practicing EBF was (1) currently breastfeeding (2) had not started giving foods other than breastmilk (3) had not given any other probed liquids or (4) semi-solid foods the previous day or night. Multivariable logistic regression was used to identify factors associated with EBF, presenting adjusted odds ratios (aOR) with corresponding 95% confidence intervals at a 5% significance level.

**Results:**

The prevalence of EBF was 62.3%. In the adjusted analysis, EBF was more common among older mothers 20–24 years, 25–29 years and 30 + years (aOR 1.4; 95% CI 1.2,1.6), (aOR 1.4; 95% CI 1.1, 1.6) and (aOR 1.3; 95% CI 1.1, 1.5) respectively compared to teenage mothers. Also, EBF was more likely among mothers who lived in rural areas compared to urban areas (aOR 1.1; 95% CI 1.0, 1.3) and those who attended antenatal care (ANC) (aOR 2.2; 95% CI 1.5, 3.1). On the contrary, EBF was less common for children aged 3–5 months compared to younger (aOR 0.5; 95% CI 0.5, 0.6) and children who had received Vitamin A supplementation (aOR 0.7; 95% 0.6, 0.8).

**Conclusion:**

The study suggests that most districts in Uganda might not have made significant strides in improving EBF rates over the last twenty years, pointing to possible ongoing hurdles that need urgent attention. Particularly, there’s a pressing need to focus on teenage mothers. Maintaining and strengthening programs that advocate EBF, such as ANC, is crucial to bridge the gaps and bring about more equitable rates among different groups.

## Background

For the first six months, the World Health Organization (WHO) and the United Nations Children’s Fund (UNICEF) recommend that an infant should be given no other food or drink – “not even water” – except breast milk i.e., exclusive breastfeeding (EBF) [1]. During this period, breast milk is not only adequate to provide all the nutritional needs of the infant but also provides additional benefits such as protection from infections and allergies, promoting adequate brain development [2]. To the mother, EBF may provide additional benefits such as weight loss and delayed ovulation leading to improved child spacing [3]. Global estimates show that only about 46% of infants are exclusively breastfed for the first 6 months of life [4]. Unlike what is typically observed for well-being indicators, EBF rates are higher in Eastern and Southern Africa (47%) compared to more developed regions such as Eastern Europe and Central Asia (24%) [5]. Cultural and economic differences, along with enabling factors in social, and governance domains, may explain this variation [6]. However, within African regions and countries, disparities in EBF are evident, lower rates are observed in Central and Western Africa (28%) [5]. Moreover, EBF rates are overestimated as they are based on cross-sectional studies that define EBF as the child receiving “nothing else but breastmilk in the 24 hours preceding the interview" [5, 7, 8]. 

In Uganda, EBF rates have averaged 63% for more than a decade [7], a proportion that is much higher than what is observed in Africa and globally [5]. However, malnutrition rates remain of much concern in the country with about 29% of the children under 5 years being stunted [7]. Stunting is a form of undernutrition that occurs from the period just before conception but is most prominent in the first 1000 days of life. For the first 6 months after birth, a child should be exclusively breastfed [2, 9]. Evidence suggests that EBF during this time may reduce stunting among children by up to 50% [10, 11]. Consequently, one of the strategies to decrease the stunting rates observed in Uganda should be diverse efforts towards having noticeable gains in improving EBF rates. This requires an understanding of factors that could be associated with EBF. Although some studies shed some light on the matter, the most comparable study is almost two decades old [8]. Other similar studies are limited in scope covering either EBF in the context of the Prevention of Mother-to-Child Transmission (PMTCT) [12] or the informal sector [13] and besides all being limited in geographical scope. Overall, such studies show that the factors influencing child nutrition, including breastfeeding, are multifaceted [14]. Such include aspects such as cultural influences [15–18], economic disparities [13, 19, 20], and sociodemographic characteristics [17, 21] among other factors. This study aimed to investigate factors linked to EBF by utilizing data obtained from a routine community-based survey conducted across 77 districts in Uganda during the 2021/22 period.

## Study design and sampling

The lot quality assurance sampling (LQAS) survey is a large-scale cross-sectional survey that provides an accurate measure of the coverage of service system quality at an aggregate level, such as at the district or regional level [17, 22, 23]. It does this by using a small sample size to make binary decisions about the quality of individual units within distinct categories or areas. This method was designed to minimize costs and resources by making localized assessments rather than comprehensive evaluations. LQAS is particularly useful in situations where resources are limited and quick decisions are needed, such as in healthcare interventions or quality control in manufacturing.

To conduct the survey, each district was divided into 5 to 7 lots (referred to as supervision areas) based on established criteria such as administrative boundaries and population attributes. The study used a probability proportional to size sampling technique, selecting either 19 or 24 villages from each designated lot. At the village level, the reference household was determined through a straightforward random sampling procedure. The initial interview was conducted with the nearest household to the reference point if respondents meeting the criteria were available. In instances where they were not, subsequent households were considered until the survey was concluded. For respondents within households, selection was accomplished through simple random sampling when multiple categories or respondents within a category were present. More information about the survey and its routine application in Uganda can be found in the following references [17, 23–27]. The 2021 and 2022 LQAS surveys covered 77 districts in Uganda in the regions of Busoga, Bugisu, Bukedi, Acholi, Lango, Ankole and Kigezi,

### Study population

The study used responses from biological mothers of children less than 6 months old, who were interviewed as part of the broader category of respondents, which was biological mothers of children 0–11 months old that are of interest to the LQAS survey.

### Study variables and measurements

EBF was the dependent variable. An infant was considered to have been exclusively breastfed if she or he was given no other food or drink – “not even water” – except breast milk besides the medical exceptions stipulated by UNICEF and WHO (.i.e proportion of infants who received breast milk only in the 24 hours) [1]. It was categorized as a binary variable (Yes = child exclusively breastfed, No = child not exclusively breastfed). To construct this indicator as accurately as possible, the study considered a mother of a child under 6 months who was (1) currently breastfeeding (2) had not started giving foods other than breastmilk (3) had not given any other probed listed liquids the previous day or night and (4) had not given any semi-solid food the previous day or night. This approach addressed potential limitations that could lead to inaccurate reporting [28]. The study was a secondary analysis; therefore, the independent variables were limited to the variables collected during the LQAS surveys. However, the study utilized the UNICEF Conceptual Framework on Maternal and Child Nutrition [14] to determine the most situated variables to pick in addition to the findings of studies elsewhere. The study incorporated several independent variables, comprising sociodemographic factors (child age, maternal age, child’s gender, maternal marital status, maternal education level, location-specific attributes, household size) and health-related factors (attendance of antenatal care (ANC), place of childbirth, utilization of modern contraceptives, pregnancy status, and maternal dietary patterns (considered as “Yes” if a mother had consumed food from at least three food groups during the day preceding the survey and otherwise “No”).

### Statistical analysis

The pooled data were analyzed using STATA version 17. Descriptive statistics were computed using frequencies and percentages for categorical variables and means and standard deviations for continuous variables. Chi-square tests were performed comparing independent categorical variables (sociodemographic factors and health-related factors) with the dependent variable (EBF which was categorized as Yes or No). Multivariable logistic regression was performed for variables that were statistically significant following the chi-square test; unadjusted and adjusted odds ratios were computed and presented with their corresponding 95% Confidence Intervals. A *p*-value of less than 0.05 was taken to be statistically significant. The variable modern contraceptive use was omitted from the multivariable regression analysis due to multicollinearity. Model testing was done to assess the model with only significant variables and the model including marginally significant variables (mother’s marital status and education level), however, these were not significant and were dropped from the final model which had only the variables significant at bivariate analysis.

## Results

### Sociodemographic factors and the prevalence of EBF

Table 1 shows the study findings on the prevalence and EBF and the sociodemographic factors associated. Overall, the study examined the responses of 7,210 mothers of children under 6 months. The mean age of the mothers was 26.1 (± 6.4) years, the mean age of the children was 2 (± 1.7) months, and the mean household size of the participants was 5.3 (± 2.5) persons. Most of the mothers were married (95.0%), attained primary education (69.6%), lived in households with less than the mean household size (63.8%), and lived in rural Uganda (80.6%). The prevalence of EBF in the 77 districts was 62.3% (95% CI 61.2–63.4). EBF was more common for younger children (0–2 months) [70.1% versus 53.7%, *p* < 0.001] compared to older, among older mothers (20–24 years, 25–29 years, and 30 + years at 63.18%, 62.9%, 62.5% respectively, *p* = 0.018) compared to young mothers (57.3%), among mothers who lived in rural areas compared to urban (63.1% vs. 58.8%, *p* = 0.034). There were no statistically significant observations made between EBF and the child’s sex, mother’s marital status, mean household size and mother’s highest attained level of education.

**Table 1:** Bivariate analysis of sociodemographic factors and EBF

Variables	Frequency	*Exclusively breastfed*		
	N = 7,210			*p* value
		No (n = 2,718)	Yes (n = 4,492)	
**Child age**				< 0.001*
**0–2 months**	3,792 (52.6)	1,134 (29.9)	2,658 (70.1)	
**3–5 months**	3,418 (47.4)	1,584 (46.3)	1,834 (53.7)	
**Child sex**				0.419
**Male**	3,535 (49.0)	1,316 (37.2)	2,219 (62.8)	
**Female**	3,675 (51.0)	1,402 (38.2)	2,273 (61.8)	
**Mother marital status**				0.066
**Unmarried**	362 (5.0)	153 (42.3)	209 (57.7)	
**Married**	6,848 (95.0)	2,565 (37.5)	4,283 (62.5)	
**Mother’s age (completed years)**				0.004*
**10–19**	999 (13.9)	427 (42.7)	572 (57.3)	
**20–24**	2,400 (33.3)	869 (36.2)	1,531 (63.8)	
**25–29**	1,735 (24.1)	644 (37.1)	1,091 (62.9)	
**30+**	2,076 (28.8)	778 (37.5)	1,298 (62.5)	
**Mother’s highest attained level of education**				0.853
**None**	381 (5.3)	141 (37.0)	240 (63.0)	
**Primary**	5,016 (69.6)	1,889 (37.7)	3,127 (62.3)	
**Secondary**	1,363 (18.9)	510 (37.4)	853 (62.6)	
**Above secondary**	450 (6.2)	178 (39.6)	272 (60.4)	
**Household size (hhs)**				0.725
**<=mean hhs**	4,597 (63.8)	1,726 (37.6)	2,871 (62.5)	
**>mean hhs**	2,613 (36.2)	992 (38.0)	1,621 (62.0)	
**Residence**				0.003*
**Urban**	1,397 (19.4)	575 (41.2)	822 (58.8)	
**Rural**	5,813 (80.6)	2,143 (36.9)	3,670 (63.1)	

N = Overall Total, n = subtotal, *Denotes statistical significance at*p* **< 0.05**

### Health-related factors and prevalence of EBF

Table 2 shows the detailed findings of the bivariate analysis of health-related factors and EBF. The practice of EBF was more likely among mothers who attended ANC compared to those who did not (62.7% versus 42.1%, *p <* 0.001), for children who had not received Vitamin A supplements (65.3%) compared to those who had (54.6%) or those whose mothers did not know the Vitamin A supplementation status (53.6%), *p <* 0.001. Also, EBF was more common among mothers who were not current users of any modern family planning method compared to those who were users (62.8% versus 42.6%) and those who were not pregnant (63.0%) compared to those who were (54.0%) and those that did not know their current pregnancy status (53.4%), *p = *0.036.

**Table 2:** Bivariate analysis of health-related factors and EBF

Variables		Exclusively breastfed		
	Frequency			*p* value
	**N = 7,210**	No (n = 2,718)	Yes (n = 4,492)	
**ANC Attendance**				< 0.001*
**No**	140 (1.9)	81 (57.9)	59 (42.1)	
**Yes**	7,070 (97.7)	2,637 (37.3)	4,433 (62.7)	
**Months at 1st ANC**				0.213
**1**	482 (6.8)	194 (40.3)	288 (59.8)	
**2**	1,054 (14.9)	390 (37.0)	664 (63.0)	
**3**	2,096 (29.6)	772 (36.8)	1,324 (63.2)	
**4**	1,687 (23.9)	601 (35.6)	1,086 (64.4)	
**5**	1,751 (24.8)	680 (38.8)	1,071 (61.2)	
**ANC attendance times**				0.142
**< 8 times**	6,402 (91.4)	2,367 (37.0)	4,035 (63.0)	
**>=8 times**	600 (8.6)	240 (40.0)	360 (60.0)	
**ANC attendance in 1st trimester**				0.948
**No**	3,438 (48.6)	1,281 (37.3)	2,157 (62.7)	
**Yes**	3,632 (51.4)	1,356 (37.3)	2,276 (62.7)	
**Delivery place**				0.665
**Home/other**	1,055 (14.6)	404 (38.3)	651(61.7)	
**Health facility**	6,155 (85.4)	2,314 (37.6)	3,841 (62.4)	
**Counselling on infant feeding**				0.455
**No**	1,857 (26.3)	706 (38.0)	1,151 (62.0	
**Yes**	5,213 (73.7)	1,931 (37.0)	3,282 (63.0)	
**Vitamin A supplementation**				< 0.001*
**No**	5,192 (72.0)	1,801 (34.7)	3,391 (65.3)	
**Don’t know**	162 (2.3)	74 (45.7)	88 (54.3)	
**Yes**	1,856 (25.7)	843 (45.4)	1,013 (54.6)	
**Member mother care group**				0.075
**No**	6,829 (94.7)	2,558 (37.5)	4,271 (62.5)	
**Yes**	381 (5.3)	160 (42.0)	221 (58.0)	
**Mother dietary diversity**				0.463
**No**	6,093 (84.5)	2,286 (37.5)	3,807 (62.5)	
**Yes**	1,117 (15.5)	432 (38.7)	685 (61.3)	
**Modern FP use**				< 0.001*
**No**	7,041 (97.7)	2,621(37.2)	4,420 (62.8)	
**Yes**	169 (2.3)	97 (57.4)	72 (42.6)	
**Currently pregnant**				0.036*
**No**	6,845 (97.4)	2,530 (37.0)	4,315 (63.0)	
**Don’t know**	73 (1.0)	34 (46.6)	39 (53.4)	
**Yes**	113 (1.6)	52 (46.0)	61 (54.0)	

N = Overall Total, n = subtotal, *Denotes statistical significance at *p* **< 0.05**

### Factors associated with EBF

Table 3 presents the multivariate analysis of factors associated with EBF. In the adjusted analysis, among the sociodemographic factors, children aged 3–5 months had 50% lower odds of EBF compared to younger children (aOR 0.5; 95% CI 0.5–0.6, *p* < 0.001). However, the odds of EBF were 40%, 40% and 30% higher among mothers 20–24 years, 25–29 years and 30 + years [(aOR 1.4; 95% CI 1.2–1.6, *p* < 0.001), (aOR 1.4; 95% CI 1.1–1.6, *p* < 0.001) and (aOR 1.3; 95% CI 1.1–1.5, *p* < 0.001) respectively compared to younger mothers (10–19 years). Additionally, EBF odds were 10% higher among mothers who lived in rural areas compared to those in urban areas (aOR 1.1; 95% CI 1.0–1.3, *p* = 0.034). Among the health-related factors, the odds of EBF were more than twice as high among mothers who attended ANC (aOR 2.2; 95% CI 1.5–3.1, *p* < 0.001), 30% less among children who had received Vitamin A supplementation (aOR 0.7; 95% 0.6–0.8, *p* < 0.001).

**Table 3:** Factors associated with EBF

Variables	Unadjusted OR (95%CI)	*P* value		Adjusted OR (95%CI)	*P* value
**Child age**					
**0–2 months**	1 (Reference)		1 (Reference)		
**3–5 months**	0.5 (0.4, 0.5)	< 0.001*	0.5 (0.5, 0.6)		< 0.001*
**Mother age**					
**10–19**	1 (Reference)		1 (Reference)		
**20–24**	1.3 (1.1, 1.5)	< 0.001	1.4 (1.2, 1.6)		< 0.001
**25–29**	1.3 (1.1, 1.5)	0.004*	1.4 (1.1, 1.6)		< 0.001*
**30+**	1.2 (1.1, 1.5)	0.005*	1.3 (1.1, 1.5)		0.001*
**Residence**					
**Urban**	1 (Reference)		1 (Reference)		
**Rural**	1.2 (1.1, 1.3)	0.003*	1.1 (1.0, 1.3)		0.034*
**ANC Attendance**					
**No**	1 (Reference)		1 (Reference)		
**Yes**	2.2 (1.6, 3.2)	< 0.001*	2.2 (1.5, 3.1)		< 0.001
**Vitamin A supplementation**					
**No**	1 (Reference)		1 (Reference)		
**Don’t know**	0.6 (0.5, 0.9)	0.004*	0.8 (0.5, 1.1)		0.098
**Yes**	0.6 (0.6, 0.7)	< 0.001*	0.7 (0.6, 0.8)		< 0.001*
**Currently pregnant**					
**No**	1 (Reference)		1 (Reference)		
**Don’t know**	0.7 (0.4, 1.1)	0.093	0.8 (0.5, 1.3)		0.299
**Yes**	0.7 (0.5, 1.0)	0.049*	0.8 (0.5, 1.1)		0.174

*Denotes statistical significance at *p* < 0.05

## Discussion

The present study investigated the prevalence of EBF in 77 districts that conducted the LQAS survey in 2021 and 2022. EBF is a globally recommended practice that aims at not only improving infant and young child nutrition but also providing additional benefits to the mother and child [1–3]. The present study findings showed that 62.3% of children under 6 months were exclusively breastfed. The child’s age, mother’s age and residence were the sociodemographic factors associated with EBF. Older mothers and mothers living in rural areas were more likely to exclusively breastfeed their children. Among the health-related factors, mothers who had attended ANC during the pregnancy of the child were more likely to exclusively breastfeed while children who had received Vitamin A supplementation were less likely to be exclusively breastfed.

The proportion of EBF observed by this study is comparable to the average observed by the 2016 Uganda Demographic and Health Survey (UDHS) (63%) more than seven years ago [7]. It is therefore likely that there have not been any significant gains in increasing the prevalence of EBF in the majority of the distrcits over the past 20 years in Uganda. Although the observed rate was still higher than most countries in Africa and globally [5], attaining greater improvements in EBF could play a vital role in preventing chronic childhood undernutrition in the country [7, 10, 11, 17]. Moreover, that older mothers were more likely to exclusively breastfeed compared to teenage mothers was like the observation made by researchers in Ethiopia [21]. Unlike teenage mothers, older mothers may have more experience and knowledge about the benefits of EBF due to previous pregnancies and motherhood. This awareness can influence their decision to breastfeed exclusively [29, 30]. Besides, older mothers may have more stable socioeconomic circumstances, which can positively impact their ability to exclusively breastfeed [13]. Understanding and addressing the causes of lower EBF rates among teenage mothers could accelerate national efforts towards improving EBF. This is particularly notable since about quarter of teenage girls in Uganda, have begun their motherhood journey, among them, 19% have already given birth, and 5% are expecting their first child [7].

Moreover, the study findings showed that mothers living in rural areas were more likely to exclusively breastfeed. Although this is unlike findings in Southwest Ethiopia [19], rural-urban gaps in breastfeeding studied in Lao highlight much lower rates among urban mothers [20]. Moreover, still unlike findings in Southwest Ethiopia [19], other studies have found that higher education among women which is common in urban women was linked to lower rates of EBF rates [31, 32]. This could be attributed to a disparity in workplace dynamics, incomes and access to breastmilk substitutes which are some of the reasons attributed to lower rates of EBF in urban areas [13, 20]. An integrated approach that supports the education and employment of women and additionally incorporates the demands of motherhood must be explored [13]. Otherwise, Uganda will persist in facing challenges related to lower EBF rates among urban mothers, contributing to a state of overall stagnation for almost a decade [7].

On the other hand, in agreement with the findings of the study in Southwest Ethiopia [19] mentioned earlier and other studies [13, 33], ANC attendance was linked to a higher likelihood of exclusively breastfeeding. ANC is an opportunity to provide education and counselling on not only EBF but also other proper infant feeding practices [19, 33, 34]. Therefore, this study underscores the importance of ANC attendance in fostering proper infant and young child nutrition practices. However, ANC attendance must be complemented by other desired health-seeking behavior such as institutional birth delivery which although not observed by this study is a studied predictor of EBF [33]. Consequently, such factors may work in tandem to foster higher rates of EBF. Moreover, consideration needs to be made for the number of times a mother attended which also predicts EBF rates [13, 19]. This is possibly because various information may be shared during the different contact visits and a mother who attended fewer visits may miss some information. The current study, however, found no significant association between the number of ANC visits and EBF.

In this study, children who had received Vitamin A supplementation were less likely to be exclusively breastfed. Typically, according to WHO recommendations, Vitamin A supplementation is advised to commence at 6 months of age [35]. This aligns with the cessation of the duration of EBF. However, when an infant under 6 months of age is not exclusively breastfed, the WHO additionally suggests Vitamin A supplementation, but not as a strong public health intervention [36]. This is due to the current evidence being less definitive, and the balance between benefits and risks being less certain. Considering the limitations of the present study, the rationale for Vitamin A supplementation in children under 6 months might be rooted in the need to assess the risk of morbidity or mortality, a question for which the available data were insufficient to provide a conclusive answer. As a result, it is somewhat surprising that some children under 6 months received Vitamin A supplementation. On the positive side, this practice may provide some breastmilk-like benefits to these non-exclusively breastfed infants, such as immune support [35, 36]. Nevertheless, it remains uncertain whether health workers administered Vitamin A based on EBF status, if Vitamin A was provided without considering the infant’s age, or if the recollection of events played a role. This ambiguity could serve as a potential avenue for future research.

This study was a secondary analysis of data from the district-based 2021 and 2022 LQAS surveys that covered more than half of the districts (77) in Uganda to present the most current findings on EBF. It benefited from its remarkable sample size that was representative of districts giving reliable estimates and robust coverage unlike similar studies in the country [13]. However, it is essential to acknowledge certain limitations inherent in the study. Firstly, the use of cross-sectional LQAS surveys, which rely on reported data regarding EBF practices, exposes the research to the inherent constraints of cross-sectional research designs and potential social desirability bias [37, 38]. For instance, this study assessed EBF among a combined sample of children 0–6 months at a point in time. However, EBF practices might vary over time, with some mothers practising it intermittently, making it challenging to precisely determine EBF although it was a, straightforward method ( reporting EBF rates for the 24 hours before the survey). As a result, the actual EBF rates could potentially be much lower. Future LQAS surveys may need to consider collecting EBF since birth to establish an even more reliable estimate. Additionally, although the researchers carefully studied variable selection for the study using the UNICEF Conceptual Framework on Maternal and Child Nutrition [14], it is important to note that this study was limited by its inability to consider certain factors due to data availability constraints.

## Conclusion

The study findings indicated a possible lack of significant progress in enhancing EBF rates in Uganda. Maternal age, residence, and ANC attendance were some of the predictors of EBF. . The limited progress in improving EBF rates over the past two decades warrants a call for more efforts to address existing barriers and the use of evidence-based findings such as provided by this study to increase EBF rates. For instance, the study found that teenage mothers were less likely to practice EBF , Uganda needs to prioritize tailored education and accessible resources to empower teenage mothers for EBF, promoting both maternal and infant health. Urban-rural disparities in EBF rates are prevalent, potentially due to differences in employment patterns. The implementation of policies and strategies around those areas need to be strengthened, particularly to address EBF beyond what the law provides or in employment contexts. For instance, the area remains weak as beyond the 60-day maternity leave, mothers may be forced to cease EBF if workplaces do not provide an enabling environment. Practices that positively influence EBF such as ANC attendance should be maintained and strengthened to bridge any gaps with a focus on the quality of ANC contact visits. Finally, future studies in Uganda should aim to estimate the EBF rates since birth to provide a more comprehensive picture and delineate the ambiguity regarding Vitamin A supplementation among children under 6 months. 

## Data Availability

The data that support the findings of this study are available upon request from Social & Scientific Systems, Inc., a DLH Holdings Company. In compliance with our institutional data-sharing policy we cannot publicly provide the data as part of the manuscript submission. To obtain the data from Social & Scientific Systems, Inc., a DLH Holdings Company, interested researchers should contact Daraus Bukenya (Dr.) at Daraus.Bukenya@dlhcorp.com, Barbara Amuron (Dr.) at Barbara.Amuron@dlhcorp.com or the DLH Institutional Review Board (IRB) at IRBHelp@dlhcorp.com. If researchers prefer to request data from specific districts, they may reach out to the corresponding district offices through the Ministry of Local Government at ps@molg.go.ug. Requests for data from Social & Scientific Systems, Inc., a DLH Holdings Company will be subject to review by the IRB Review Team, ensuring adherence to ethical and legal considerations.

## Abbreviations

ANC: Antenatal care
aOR: Adjusted odds ratio
EBF: Exclusive breastfeeding
hhs: Household size
HIV: Human immunodeficiency virus
LQAS: Lot quality assurance sampling survey
PMTCT: Prevention of Mother-to-Child Transmission
SSA: Sub-Saharan Africa
SD: Standard deviation
uOR: Unadjusted odds ratio
UDHS: Uganda Demographic and Health Survey
UNICEF: The United Nations Children’s Fund
WHO: World Health Organization
